# Impact of Infection Control Interventions on Candida auris at a Tertiary Care Center in Bahrain: A Five-Year Experience

**DOI:** 10.7759/cureus.105859

**Published:** 2026-03-25

**Authors:** Saleh F Sowar, Rommel Acunin, Harold C Cabanalan, Safa Alkhawaja, Mohamed A Dakheel, Hassan I Darwish, Jumana A Ahmed, Athraa S Naser

**Affiliations:** 1 Infection Control, Government Hospitals, Manama, BHR; 2 Internal Medicine, Government Hospitals, Manama, BHR

**Keywords:** antifungal stewardship, bahrain, candida auris, environmental disinfection, hospital‑acquired infections, infection control

## Abstract

Introduction

*Candida auris* (*C. auris*) is a multidrug-resistant fungal pathogen associated with high morbidity, environmental persistence, and rapid transmission in healthcare settings. This study evaluated the impact of a comprehensive infection-control intervention on hospital-acquired *C. auris* cases by comparing two post-implementation periods, phase I (2021-2022) and phase II (2023-2025), to assess the intervention’s performance over time.

Methods

A retrospective analysis was conducted on 323 patients diagnosed with *C. auris* between 2021 and 2025. Infection-control measures were initiated at the end of 2021 as part of the institutional response to the detection of the initial cases and were consistently maintained until the end of the study. Chi-square tests were applied to compare demographic and clinical characteristics across the two periods.

Results

The majority of patients with *C. auris* were male (65.3%), and more than half (51.4%) were over 65 years of age. Demographic and clinical characteristics were similar between the two periods. However, the proportion of patients with documented contact with a *C. auris*-positive individual significantly decreased over time, from 56.9% in phase I (2021-2022) to 40.8% in phase II (2023-2025) (*p* = 0.004). Monthly hospital-acquired *C. auris* cases decreased from 6 cases per month in phase I to 5 cases per month in phase II, representing a 16.7% reduction; however, this difference was not statistically significant (p = 0.41).

Conclusion

The multidisciplinary infection-control intervention significantly reduced contact-associated transmission and hospital-acquired *C. auris* cases. The results show that multidisciplinary collaboration, improved screening, and enhanced environmental cleaning and disinfection all help control *C. auris* in high-risk healthcare settings.

## Introduction

*Candida auris* is a multidrug-resistant fungus that can cause severe illness, particularly in hospitalized and immunocompromised individuals. First identified in 2009 in Japan, *C. auris* has attracted increased attention as a global threat since the COVID-19 pandemic because of reported regional and global surges in incidence and outbreaks associated with high mortality rates among hospitalized patients [1,2]. It is more frequently transmitted within healthcare facilities because of challenges in infection control measures, particularly hand hygiene, contact precautions, and environmental cleaning and disinfection. Even in healthcare settings with sound infection prevention and control systems, transmission and outbreaks of this pathogen can occur [3]. Common infection prevention and control challenges reported with *C. auris* include difficulty diagnosing infections using standard microbiology methods, an exceptional ability to adhere to and survive on environmental surfaces for several weeks, resistance to common hospital disinfectants and standard antifungal treatments, and long-term colonization of infected patients [4,5].

A multipronged infection prevention and control approach is necessary to halt the transmission of *C. auris* in healthcare settings. These measures include active screening among high-risk patients, standard and contact isolation precautions, and intensified cleaning and disinfection of hospital environmental surfaces. Any lapses in these measures may increase the incidence of healthcare-associated *C. auris* [6,7].

Worldwide, *C. auris* remains a significant concern in healthcare delivery. In Bahrain, data remain limited regarding the incidence and effective management of *C. auris* outbreaks [8]. Despite several challenges and resource limitations, our institution has adopted established guidelines to reduce the incidence of healthcare-associated *C. auris* in our facility. Thus, this study discusses the infection prevention and control strategies used to effectively manage *C. auris* at Bahrain’s largest governmental tertiary-care hospital.

In the Gulf Cooperation Council (GCC) region, *C. auris* remains a concern and poses a significant threat to healthcare facilities across the region [8]. COVID-19 further increased the risk of healthcare-associated infections, as multiple studies have reported that *C. auris* increased markedly during the pandemic. This may be due to numerous factors, including increased ICU admissions, use of invasive devices, antimicrobial use, and significant strain on hospital resources (e.g., severe shortages of personal protective equipment, staffing, and workload demands) [1,9,10]. Despite this, effective infection prevention and control interventions for *C. auris* are broadly similar across continents.

Prevention and control of *C. auris* are well established in the literature, and healthcare organizations have adopted successful interventions. Ahmad S and Asadzadeh M (2023) and Caceres DH et al. (2019) emphasized that rapid and accurate identification of *C. auris* is paramount for infection prevention and is the most crucial step in preventing further transmission and potential outbreaks within healthcare facilities [11,12]. To ensure rapid detection of *C. auris*, developing clear screening guidelines and protocols is a priority. According to Alshamrani MM et al. (2020), active screening followed by stringent infection prevention and control measures is the key strategy for successfully controlling *C. auris* outbreaks [13]. Cheng A et al. (2025) highlighted the effectiveness of expanded screening programs during hospital admission for early detection of cases and prevention of potential intra-hospital transmission [14]. This was also emphasized by Kenters N et al. (2019) during the International Society for Antimicrobial Chemotherapy expert meeting regarding *C. auris* in healthcare [15]. In an investigation of a large outbreak of *C. auris* at a quaternary hospital in Saudi Arabia, it was found that 60% of cases represented colonization and approximately 40% represented infection. Moreover, data suggest that the majority of these cases, including reports from the UAE, are hospital-acquired [16,17].

For recommended hand hygiene practices, Alfouzan WA et al. (2022) suggest that performing hand rubbing with an alcohol-based hand rub after hand washing with soap and water, to ensure maximal hand disinfection, can be considered a major prevention strategy [18]. Strict contact precautions have also been considered the gold standard for managing *C. auris*, with an approach similar to that used for other contact-transmissible organisms. Contact precautions are applied for confirmed cases and those exposed with pending screening results [11,16,19]. Several authors have also explored the use of chlorhexidine bathing to reduce *C. auris* bioburden on patients’ skin [18]. Lee EH et al. (2024) reported that chlorhexidine bathing was part of their aggressive strategy to address a large-scale outbreak in the ICU [20]. However, Ku TS et al. (2018) reported persistent colonization even after twice-daily application of the solution in affected patients [21]. *C. auris* can also persist in the environment for extended periods; therefore, meticulous attention to cleaning and disinfection is critical. Many published studies report that chlorine-based solutions are the disinfectant of choice for *C. auris*, based on comparisons of the microbiocidal activity of different chemical agents against *C. auris* [15,21,22]. As an adjunct disinfection procedure, Reedy JM et al. (2024) investigated the efficacy of using ultraviolet-C (UVC) light in a room occupied by *C. auris* patients. According to the study, UVC light was 99.9% effective at inactivating *C. auris* [23].

Guidelines and recommendations vary regarding the duration of contact precautions for patients known to be colonized with *C. auris*. Some authors suggest that contact precautions may be discontinued if the patient has two consecutive negative screening results collected at least one week apart, provided the patient has been off antifungal therapy for at least one week, while Vuichard-Gysin D et al. (2020) recommend five consecutive negative results collected one week apart and keeping the patient flagged for one year after the first negative result [19,24]. Griffith N and Danziger L (2020) stated that the decision to discontinue precautions has to be made on a case-by-case basis [25]. In contrast, a renowned organization such as the CDC recommends continuing contact precautions throughout the patient’s hospitalization [26]. In the context of antimicrobial stewardship programs, Alagha R et al. (2024) highlight their importance as a major component of infection prevention and control strategies to combat the emergence of *C. auris* in Bahrain [27].

More importantly, establishing a multidisciplinary team comprising infection control, nursing, environmental services, microbiology, and hospital administration could significantly enhance facility preparedness and efficiency in controlling *C. auris* transmission within healthcare organizations [28].

Rationale

The findings of this study are important for public health and health policy in Bahrain to protect patients and help the system respond to new health threats. By evaluating *C. auris* incidence after the implementation of infection control measures, the results show that well-implemented, evidence-based prevention programs can reduce hospital-acquired *C. auris* infections and colonization, which highlights the need for ongoing investment in infection prevention, including continuous education for the infection control team, provision of advanced technologies for environmental cleaning and disinfection, and rapid *C. auris* diagnostic tools. Policymakers can use this information to improve national guidelines, use resources more effectively, and address *C. auris* as a serious public health concern that requires a coordinated response. To our knowledge, limited studies have been conducted in Bahrain on the prevention of *C. auris*; hence, this study aimed to evaluate the prevention and control of *C. auris* cases at a major tertiary care center in Bahrain between 2021 and 2025.

## Materials and methods

This study used a retrospective, observational, comparative, and record-based design to examine patients with *C. auris* infection or colonization admitted to the Salmaniya Medical Complex (SMC), a branch of Government Hospitals (GHs) in Bahrain, from January 2021 to December 2025. All data were obtained exclusively from SMC, the largest government tertiary care hospital in the Salmaniya district of Manama, established in 1957, with about 1,200 beds. It is the largest referral center in Bahrain, offering secondary, tertiary, emergency, and specialized outpatient services. Data were collected retrospectively from the hospital database, and all cases that met the inclusion criteria were included.

Sample size estimation and sampling method

A formal sample size calculation was not performed because the study used a census sampling approach, in which all eligible hospital-acquired *C. auris* cases recorded at SMC from January 2021 to December 2025 were included. Census sampling is appropriate for retrospective surveillance studies in which the entire target population is accessible. This study included all patients of any age or gender who had a laboratory-confirmed *C. auris* infection or colonization and met the definition of a hospital-acquired case, which required the positive sample to be collected on or after the third calendar day of admission. Only cases with complete demographic, clinical, and microbiological information were eligible for inclusion. Community-acquired cases, defined as those with a positive sample collected before day three of hospitalization, were excluded. We also excluded suspected but unconfirmed cases, incomplete or missing records, duplicate entries except for the first valid record, and diagnoses outside the study period. This process helped ensure that the final dataset included only confirmed hospital-acquired *C. auris* cases with complete and reliable information for analysis.

Infection control prevention method

Specific infection prevention and control measures for *C. auris* in GHs include activating a multidisciplinary task force to manage *C. auris*, involving infection control, microbiology, nursing, environmental services, and hospital administration. Extensive screening of high-risk populations (those exposed to a positive case, upon admission to the adult ICU, transferred from another healthcare facility, previously admitted patients upon readmission, and before transfer to a long-term care facility) was performed for rapid identification of cases, along with placement of positive cases in a dedicated ward under strict contact precautions until discharge, and a comprehensive environmental cleaning and disinfection program, which included assigning dedicated trained environmental services staff, terminal disinfection using Environmental Protection Agency (EPA)-approved chlorine-based chemical disinfectant solutions combined with UV-C disinfection, followed by thorough inspection by the infection control team.

Screening for *C. auris*

The *C. auris* screening protocol began in 2021 and was updated as needed. Screening focused on high-risk groups, such as exposed roommates of confirmed cases, adult ICU admissions, previously admitted patients upon readmission, and patients transferred from or before transfer to other healthcare or long-term care facilities, to ensure rapid case identification [13,18]. In 2023, the routine screening protocol for healthcare workers, obstetrics and gynecology patients, and pediatric patients was discontinued because no cases were found in these groups during the initial two years.

Case identification and eligibility criteria

**Figure 1:**
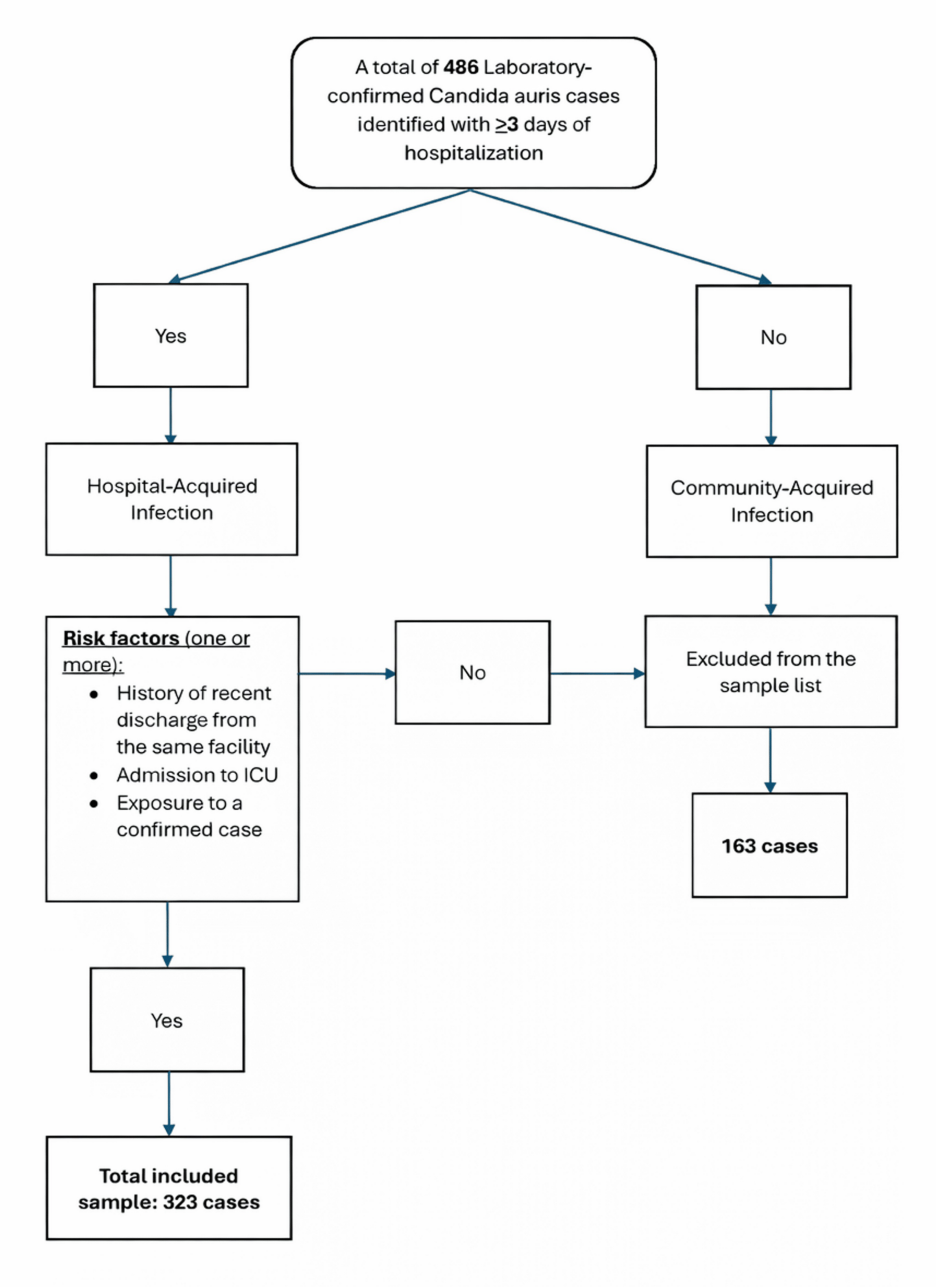
Census sampling and recruitment process.

In this study, we first screened laboratory-confirmed *C. auris* cases identified at SMC through routine culture followed by species-level confirmation using Matrix-Assisted Laser Desorption/Ionization Time-of-Flight (MALDI-TOF; Bruker Corporation, Billerica, MA, US) mass spectrometry, to identify patients who had been hospitalized for at least three days, resulting in 486 possible cases. We then classified these as either hospital-acquired or community-acquired infections. Community-acquired cases (n=163) were excluded from further analysis. For the hospital-acquired cases, we checked for specific risk factors: recent discharge from the same facility, admission to an intensive care unit, or documented exposure to a confirmed case. Only patients who met at least one of these criteria were included. After this process, 323 cases met the eligibility requirements and were included in the final analysis. This method helped ensure that our study group accurately represented patients with clear healthcare-associated exposure, in line with epidemiologic standards for infectious disease research (Figure 1).

Ethical approval

This retrospective study received approval from the Research Committee for Government Hospitals of Bahrain (research approval serial no. 125-301225) on December 30, 2025. Medical Record Numbers (MRNs) were accessed solely to retrieve the relevant patient charts, including laboratory results and patients' admission history. All identifiers were removed immediately after data extraction, and the final dataset used for analysis contained only anonymized information. The study involved no direct patient contact, and no informed consent was required.

Data collection

Data were collected and logged into the infection control surveillance system from the hospital's electronic medical records. All *C. auris*-positive cases were listed, including details such as age, gender, site of positivity, infection status (clinical infection or colonization), history of ICU admission, recent hospitalization, and contact with a confirmed case. The main intervention studied was the infection control program started in 2021. Incomplete or missing patient data were excluded from the overall dataset.

Statistical analysis

To evaluate the effectiveness of the implemented infection-control interventions, the study period was divided into two phases: phase I (2021-2022) and phase II (2023-2025). Categorical variables were described as counts and proportions (%). The comparison of phase period (phase I vs. phase II) by demographic characteristics and patients' clinical outcomes was examined using the Chi-square test or Fisher's exact test, whenever appropriate. Case trends were analyzed to assess the intervention's impact on *C. auris* incidence over a 5-year period. A p-value of less than 0.05 was considered statistically significant. All statistical data were analyzed using the SPSS, version 26 (Armonk, NY: IBM Corp., USA).

## Results

A total of 323 patients with confirmed *C. auris* colonization or infection were included in the analysis. The demographic and clinical characteristics of the cohort are summarized in Table 1. Slightly more than half of the patients were aged 65 years or older (51.4%), and males accounted for the majority of cases (65.3%). The most frequently identified positive sites were the groin (28.5%), axilla (23.5%), and urine (19.2%). Colonization was more common than clinical infection (54.2% vs. 45.8%), and nearly half of the cohort had a history of ICU admission (48.9%). Additionally, 48.0% of patients had documented contact with a *C. auris*-positive individual, and nearly half (49.5%) had been hospitalized for more than 30 days prior to diagnosis.

**Table 1:** Demographic and clinical characteristics of the patients (n = 323). Results are expressed as numbers and percentages, n (%). Data represent original hospital-acquired *Candida auris* cases from Salmaniya Medical Complex analyzed under institutional approval from the Government Hospitals Research Committee (approval serial no. 125-301225).

Study variables	N (%)
Age group	
≤65 years	157 (48.6%)
>65 years	166 (51.4%)
Gender	
Male	211 (65.3%)
Female	112 (34.7%)
Positive sites	
Axilla	76 (23.5%)
Blood	52 (16.1%)
Groin	92 (28.5%)
Deep tracheal aspirate	23 (7.1%)
Wound	8 (2.5%)
Urine	62 (19.2%)
Others	10 (3.1%)
Infection status	
Clinical sample	148 (45.8%)
Colonization	175 (54.2%)
History of ICU admission	
No	165 (51.1%)
Yes	158 (48.9%)
History of contact with positive *C. auris*	
No	168 (52.0%)
Yes	155 (48.0%)
Length of hospitalization before diagnosis with *C. auris*	
≤30 days	163 (50.5%)
>30 days	160 (49.5%)

**Figure 2:**
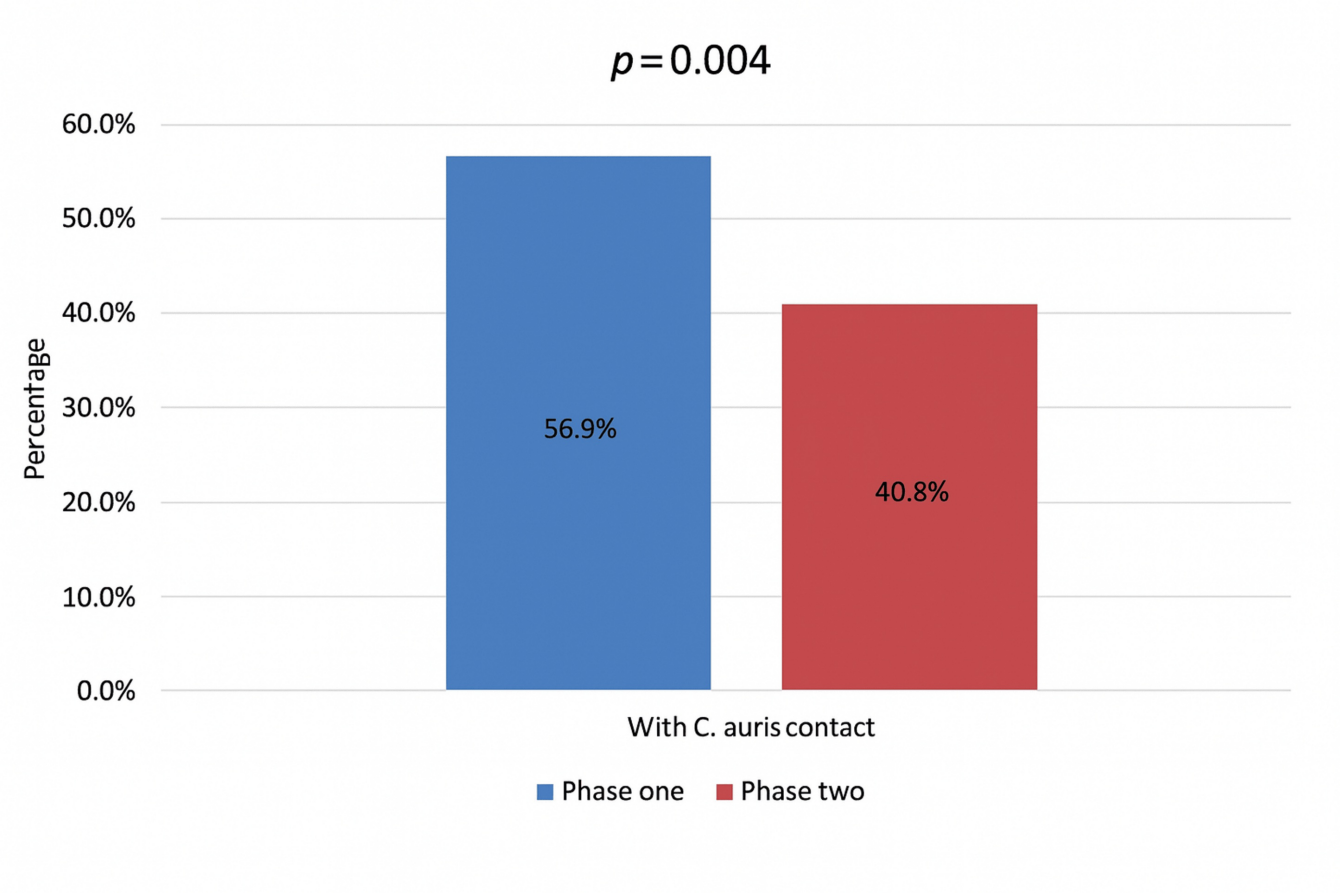
Effectiveness of the Candida auris intervention among patients with previous contact with C. auris. Comparison of contact-associated *C. auris* exposure between phase I and phase II.

A comparison of phase I and phase II periods is presented in Table 2. Most demographic and clinical variables, including age group (p = 0.073), gender (p = 0.163), distribution of positive sites (p = 0.622), infection status (p = 0.371), ICU admission history (p = 0.096), and hospitalization duration before diagnosis (p = 0.411), did not differ significantly between the two periods. This stability indicates that the intervention did not alter the underlying patient profile or case mix. However, a significant reduction was observed in the proportion of patients with a documented history of contact with a *C. auris*-positive individual. During phase I, 56.9% of patients reported such contact, compared with 40.8% in phase II (p = 0.004) (Figure 2). Although the proportion of patients with clinical infection versus colonization did not change significantly between phases, this does not contradict the observed reduction in contact-associated exposure, as *C. auris* transmission can occur through multiple pathways, including environmental contamination and indirect contact, which were not individually measured in this study. This finding suggests that the infection-control measures effectively disrupted transmission pathways and reduced exposure-related risk.

**Table 2:** Comparison of Candida auris between phase I and phase II according to the demographic and clinical characteristics of the patients (n = 323). Results are expressed as numbers and percentages, n (%). ‡ P-value was calculated using Fisher’s exact test. § P-value was calculated using the Chi-square test. ** Significant at the p < 0.05 level. Data represent original hospital-acquired *Candida auris* cases from Salmaniya Medical Complex, analyzed under institutional approval from the Government Hospitals Research Committee (approval serial no. 125-301225).

Factor	*Candida auris *intervention phase		P-value ^§^
	Phase I (2021-2022) N (%) ^(n = 144)^	Phase II (2023-2025) N (%) ^(n = 179)^	
Age group			
≤65 years	62 (43.1%)	95 (53.1%)	0.073
>65 years	82 (56.9%)	84 (46.9%)	
Gender			
Male	100 (69.4%)	111 (62.0%)	0.163
Female	44 (30.6%)	68 (38.0%)	
Positive sites			
Axilla	33 (22.9%)	43 (24.0%)	0.622^ ‡^
Blood	19 (13.2%)	33 (18.4%)	
Groin	45 (31.3%)	47 (26.3%)	
Deep tracheal aspirate	9 (6.3%)	14 (7.8%)	
Urine	27 (18.8%)	35 (19.6%)	
Wound	5 (3.5%)	3 (1.7%)	
Other	6 (4.2%)	4 (2.2%)	
Infection status			
Clinical sample	62 (43.1%)	86 (48.0%)	0.371
Colonized	82 (56.9%)	93 (52.0%)	
History of ICU admission			
No	81 (56.2%)	84 (46.9%)	0.096
Yes	63 (43.8%)	95 (53.1%)	
History of contact with positive *C. auris*			
No	62 (43.1%)	106 (59.2%)	0.004 **
Yes	82 (56.9%)	73 (40.8%)	
Length of hospitalization before diagnosis with *C. auris*			
≤30 days	69 (47.9%)	94 (52.5%)	0.411
>30 days	75 (52.1%)	85 (47.5%)	

**Figure 3:**
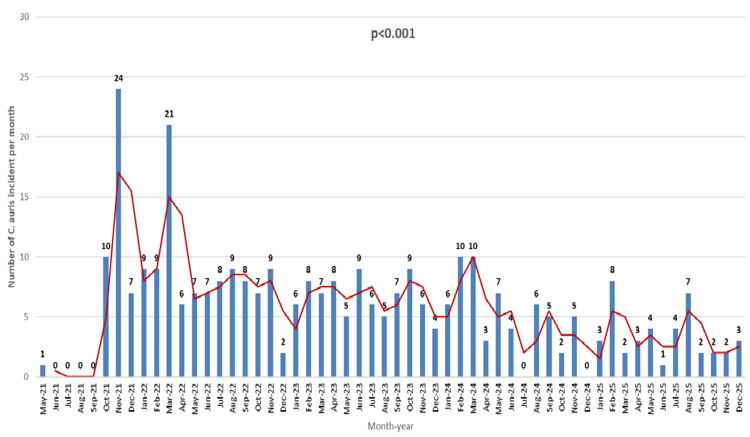
Number of hospital-acquired Candida auris cases per month from 2021 to 2025.

Figure 3 shows the monthly trend in hospital-acquired *C. auris* cases from 2021 to 2025, with a clear, statistically significant decline after the infection-control intervention was implemented (p < 0.001). In 2021 and early 2022, during the peak of the COVID-19 pandemic, the facility experienced significant fluctuations, including several sharp spikes, particularly in November 2021 (24 cases) and March 2022 (21 cases). In 2023, case spikes began to decline, and monthly case numbers stabilized, with some months reporting zero hospital-acquired cases. By the end of the study period (2025), *C. auris* cases remained consistently low. This steady drop in cases in the later years of the study highlights how effective the intervention bundle was in stopping transmission and preventing new cases.

## Discussion

Global and regional context of *C. auris*

*Candida auris* remains a major concern for infection control worldwide because it spreads quickly, is resistant to many drugs, and survives in the environment. International reports show that cases increased during the COVID-19 pandemic, likely due to crowded ICUs, greater use of medical devices, increased exposure to antimicrobials, and limited healthcare resources [1,9,10]. This center exhibited comparable trends, with older adults, intensive care patients, and individuals experiencing prolonged hospitalizations being disproportionately affected, consistent with established risk profiles in the literature [5-7]. In the GCC region, recurrent outbreaks and persistent colonization underscore the need for robust, coordinated infection-control strategies [8]. Studies from Saudi Arabia and the United Arab Emirates also show that most *C. auris* cases are acquired in hospitals, and colonization often precedes infection [16,17].

Impact of infection‑control intervention 

In 2021, our institution implemented a comprehensive multidisciplinary infection control intervention focused on *C. auris*. We followed international guidelines, such as those from the CDC and the Association for Professionals in Infection Control and Epidemiology (APIC), and adapted them for our busy tertiary care institution [11-13]. A team composed of infection control, microbiology, nursing, environmental services, and hospital administration was established to manage the outbreak and respond quickly. We screened high-risk groups upon admission to the adult ICU, patients exposed to confirmed cases, and those transferred from other healthcare facilities to identify and contain cases early. Patients who tested positive were placed in a dedicated ward with strict contact precautions until discharge, thereby reducing the risk of transmission. We improved cleaning protocols by assigning specially trained staff, using EPA-approved chlorine-based solutions in combination with UV-C room disinfection for terminal cleaning and disinfection, and conducting follow-up inspections by the infection control team. The most striking outcome of the intervention was the significant reduction in patients with documented contact with a *C. auris*-positive patient, from 56.9% in phase I to 40.8% in phase II (p = 0.004). The results show that the intervention successfully disrupted transmission pathways. Similar results have been reported in New York, Saudi Arabia, and Qatar, where improved screening, isolation, and environmental cleaning also lowered secondary transmission [14-16]. These findings highlight the importance of early identification, strict contact isolation, and environmental hygiene, which are widely recognized as key components of *C. auris* containment in the global literature [17-20].

Environmental control and disinfection challenges

One of the main challenges with *C. auris* is its ability to survive on surfaces and its resistance to many common disinfectants, as shown in earlier studies [21-22]. The use of effective disinfectants, such as chlorine-based products, along with UV-C disinfection, is now regarded as beneficial in settings with a high risk of infection [23]. These procedures are based on CDC guidelines and international expert advice. They recommend thorough cleaning, regular monitoring, and the use of disinfectants with documented effectiveness against *C. auris* [24-26]. The infection control team evaluates environmental cleaning and disinfection practices to ensure compliance with the policy; however, these audit findings were part of the overall intervention process and were not individually analyzed as outcome measures in this study.

Environmental persistence and biofilm-associated resistance of *C. auris*

*C. auris* can survive for long periods in healthcare settings because it forms strong biofilms on many types of surfaces. These biofilms, composed of polysaccharides, proteins, and lipids, help the fungus adhere to plastics, bed rails, and medical equipment and create a protective layer that makes it harder for disinfectants to work [1]. Research shows that *C. auris* biofilms are much less affected by quaternary ammonium compounds and other common disinfectants. This adaptation enables the fungus to persist on dry surfaces and withstand routine cleaning procedures [29]. This biofilm-associated resilience substantially contributes to ongoing transmission within healthcare settings, especially in high-risk units such as ICUs, where environmental contamination may serve as a reservoir for recurrent colonization and infection [30]. These observations highlight the need for improved environmental cleaning protocols, including the implementation of disinfectants with demonstrated anti-biofilm efficacy and supplementary technologies such as UV-C disinfection, to effectively disrupt transmission pathways.

Overall interpretation and implication

Overall, the findings of this study demonstrate that although the demographic and clinical characteristics of affected patients remained largely unchanged across the study period, the implementation of a comprehensive multidisciplinary infection-control strategy in 2021 substantially altered the transmission dynamics of *C. auris* within the institution. The intervention led to a marked reduction in contact-associated transmission and a sustained decline in hospital-acquired cases, reflecting the effectiveness of enhanced screening, strict contact precautions, and strengthened environmental disinfection practices. Notably, the impact of the intervention became more pronounced during 2023-2025 compared with 2021-2022, a pattern that may be attributable to the lingering operational pressures of the COVID-19 pandemic during the early implementation phase. During periods of high patient acuity, staffing constraints, and fluctuating bed occupancy, full adherence to infection-control measures may have been challenging, and this operational variability represents a potential source of bias in interpreting the intervention’s impact. As these pressures stabilized, the intervention was applied more consistently, resulting in clearer and more sustained improvements. These findings underscore that even in high-incidence, resource-constrained settings, well-structured, evidence-based, and consistently implemented infection-control programs can significantly reduce *C. auris* transmission and strengthen institutional preparedness.

Recommendations

Healthcare teams can better control *C. auris* by implementing evidence-based multidisciplinary infection control interventions. Infection control programs need to remain active and support teamwork among administrative leaders, microbiology, frontline healthcare workers, environmental services, and the infection control team. Standardizing national *C. auris* prevention and surveillance in Bahrain and the GCC can improve cooperation, early detection, faster reporting, and minimize spread between facilities, especially during outbreaks. Implementation of a risk-adjusted screening protocol to detect and isolate cases early is highly recommended. Adding regular molecular typing to surveillance can help track how the fungus spreads and distinguish ongoing environmental strains from new ones. Facilities can improve cleaning by using advanced disinfection methods such as UV-C light, vaporized hydrogen peroxide, or chlorine-based disinfectants. In our setting, chlorine-based disinfectants and UV-C were part of the intervention bundle; however, their individual contribution to the reduction in *C. auris* cases was not evaluated separately in this study. Environmental monitoring should be stepped up with regular checks and reviews of cleaning practices, especially in high-risk areas. Antifungal stewardship programs should focus on using antifungals wisely to lower the risk of resistance. Regular staff training and competency assessments help teams adhere to contact precautions, maintain cleaning routines, and identify high-risk patients early. Routine screening for healthcare workers and for low-risk populations, such as pediatric and obstetrics and gynecology patients, is not recommended.

Study limitations

This study has several limitations that should be considered when interpreting the findings. First, as a single-center analysis, the results may not be generalizable to other healthcare settings with different patient populations, resource levels, or infection control infrastructures. Second, this retrospective study relies on the accuracy and completeness of existing medical records, which may introduce documentation bias. Third, other factors, such as changes in hospital occupancy, staffing, or local epidemiology, may also have affected the transmission rate during the study period. Fourth, this study did not include molecular typing, such as whole-genome sequencing (WGS), which limits the ability to distinguish persistent environmental strains from newly introduced community strains. Without genotyping, we cannot conclusively determine whether reductions in cases reflect interruption of transmission chains or changes in strain dynamics. Finally, factors known to influence hospital-acquired transmission, such as environmental contamination, indirect contact, device use, and patient-level clinical risk factors, were part of the overall infection-control context but were not evaluated individually in this study.

Future directions

Future research should use molecular typing techniques to clarify transmission pathways and distinguish between environmental persistence and new introductions. Multicenter surveillance studies in Bahrain and throughout the GCC region are necessary to enhance understanding of national epidemiologic trends. Prospective studies evaluating the effectiveness of specific interventions, such as UV-C disinfection, targeted screening, and antifungal stewardship, may inform improvements in infection-control policies. Further research on patient outcomes, environmental contamination, and cost-effectiveness would provide valuable data for policymakers and healthcare administrators.

## Conclusions

This five-year study shows that healthcare systems face major challenges in managing *C. auris*, particularly during outbreaks or pandemics. During the study period, *C. auris* disproportionately affected vulnerable populations, including older adults, ICU patients, and individuals with extended hospital stays. After a comprehensive infection-control program was put in place in 2021, hospital-acquired *C. auris* cases decreased over time. The program also led to a clear reduction in contact-related exposure, indicating better control of direct transmission and maintaining very low case numbers by the end of phase II. These findings suggest that using proven, team-based infection-control methods can help reduce the risk of transmission, even in high-risk settings. Since *C. auris* remains a global concern, ongoing research, surveillance, and investment in prevention are essential. Additionally, this study shows that following established infection-control measures helps protect patients and healthcare workers in high-risk settings.
